# Effects of Proximity between Companion Dogs and Their Caregivers on Heart Rate Variability Measures in Older Adults: A Pilot Study

**DOI:** 10.3390/ijerph17082674

**Published:** 2020-04-13

**Authors:** Heidi K. Ortmeyer, Leslie I. Katzel

**Affiliations:** 1 Geriatric Research Education Clinical Center, VA Maryland Health Care System, Baltimore, MD 21201, USA; lkatzel@som.umaryland.edu; 2 Department of Medicine, University of Maryland School of Medicine, Baltimore, MD 21201, USA

**Keywords:** companion dogs, One Health, proximity tagging, quality of life, heart rate variability, physical activity

## Abstract

Heart rate variability (HRV) is a noninvasive tool used to evaluate autonomic nervous system function and is affected by age, stress, postural changes, and physical activity. Dog ownership has been associated with higher 24-hr HRV and increased physical activity compared to nonowners. The current pilot study was designed to evaluate the effects of proximity to a dog in real time (minute-by-minute) on older dog caregivers’ HRV measures and stress index during normal daily life over a 24-hr period. Eleven caregivers (56–83 years of age) wore ActiGraph GT9X Link accelerometers and camntech electrocardiogram monitors, and 11 dogs wore PetPace Collars and ActiGraph monitors to determine (a) proximity (absence or presence of Received Signal Strength Indicator, RSSI), (b) heart rate and HRV measures, (c) position (lying vs. sitting vs. standing), and (d) physical activity in the 11 dyads. Twenty-four hour HRV (SDNN index) and physical activity in the caregivers and dogs were related. Stress index was lower, and HRV parameters (SDNN, rMSDD, high frequency power (HF)) were higher when an RSSI signal was detected (presence of dog) compared to no RSSI signal (absence of dog) in the caregivers while inactive (lying + sitting + standing combined). HRV parameters (rMSDD and HF) were lower in the caregivers while standing and sitting compared to lying. The results from this pilot study support the hypothesis that spending time in the presence of a companion dog increases caregivers’ HRV throughout the day and suggest that proximity to a dog may contribute to overall improvements in 24-hr HRV and cardiac health in dog caregivers.

## 1. Introduction

Heart rate variability (HRV) is a useful and non-invasive biomarker of intrinsic autonomic cardiac activity [1], psychological health, and stress [2]. Reduced resting HRV indicates autonomic dysfunction and is a risk factor for metabolic disorders and cardiovascular disease mortality [3]. HRV decreases with age [4] and with prolonged sitting [5], and both long-term [6] and short-term sedentary behaviors [7] have been associated with reduced HRV. Results from the Cardiovascular Health Study, in which serial longitudinal measures of 24-hr HRV over five years in 985 older adults were evaluated, showed greater total leisure-time activity, walking distance, and walking pace were associated with more favorable HRV [8]. Older individuals had higher HRV while walking an unknown friendly dog compared to walking without a dog [9].

Friedmann et al. showed that dog ownership made a significant contribution to one-year survival after myocardial infarction [10] and higher 24-hr and daytime HRV in dog owners vs. nonowners in patients with healed myocardial infarcts [11]. In individuals with lifestyle-related diseases, pet owners had higher 24-hr, day, and night HRV compared to nonowners [12]. The presence of a dog was associated with lower systolic and diastolic blood pressure during the owner’s normal daily routine [13]. Motooka et al. reported higher HRV in four senior citizens while interacting with unknown friendly dogs in their home compared to no interaction [9]. These studies, although not all studies (e.g., [14]), suggest that dog ownership and proximity to an unknown, albeit friendly dog, are associated with beneficial effects on cardiovascular health and may contribute to healthy aging.

To date, no studies have used proximity tagging technology to determine the effects of human-animal interaction on humans’ health outcomes. We recently reported the effects of closeness and caregivers’ presence on dogs’ health outcomes using minute-by-minute proximity tagging [15]. This study was novel in several respects, notably measuring multiple outcomes (including HRV) and predictors/covariates minute-by-minute simultaneously in freely moving dogs over 10–15 days. A recent review by experts in human-animal interaction specified the need to simultaneously examine owners’ and pets’ interactions on biomarkers to provide a better understanding of their impact on healthy aging [16]. This recommendation is in harmony with the One Health approach to animal-assisted interventions, which recognizes that the health of people is connected to the health of animals and the environment, and aims to ensure that added value or synergistic benefits can be achieved in both members of the dyad [17,18]. The use of an ActiGraph Link monitor in conjunction with an electrocardiogram (ECG) monitor (human) and PetPace Collar (dog) allows for continuous and simultaneous HRV, proximity (human–dog dyad), activity, and body position data collection during a daily normal life in both the human and dog.

The purpose of this pilot study was three-fold. Our first aim was to determine whether a non-invasive method to measure the effects of minute-by-minute proximity on HRV parameters could be used to examine the interconnection and mutual benefits between humans and dogs. Our second aim was to determine whether time spent in proximity between a caregiver and their dog would affect HRV throughout the day in both members of the dyad. Our last aim was to determine whether 24-hr HRV and physical activity were related within the human–dog dyad.

## 2. Materials and Methods

Study procedures were approved by the University of Maryland Institutional Research Board (HP-00074763) and Institutional Animal Care and Use Committee (HKO-061701A), as well as the Veterans Affairs Research & Development Committee (1200930). Foster caregivers were recruited from two local dog rescue groups for the 24-hour study; all participants signed informed consent. Eligibility included > 55 years of age and participants agreed to be home alone (no other humans living in home) for at least 80% of the 24-hr period. The participants kept a log for the 24-hr period to indicate sleep times, when they were away from the home, and when they were interacting with another human (visitor) in their home. The participants completed the Dog Owner Specific Quality of Life (DOSQOL) questionnaire [19], modified for foster caregivers [20], at the end of the study period. The participant’s weight and height (self-reported) were used to calculate body mass index (BMI).

Human participants wore a Bluetooth-enabled Actigraph GT9X Link (ActiGraph, Pensacola, FL, USA) accelerometer on their thigh to measure physical activity (100 Hz sample rate), position, and proximity (receiver) (Received Signal Strength Indicator, RSSI) (100 Hz sample rate). An Actiwave Cardio (256 Hz sample rate) or ActiHeart 5 ECG (512 Hz sample rate) monitor (camntech, Boerne, TX, USA) was worn on the chest via two standard ECG electrodes. The accelerometer function on the ECG monitors was set to 25–100 Hz sample rate.

Dog participants wore an Actigraph GT9X Link accelerometer on their collar to measure physical activity (100 Hz sample rate) and proximity (beacon) and a PetPace Smart-collar to collect activity, position, pulse, and vasovagal tonus index (VVTI) at a sample rate of every two minutes as previously described [15]. Data were anaalyzed via ActiLife v6.13.4 and the PetPace Health Monitoring System. PetPace and ActiGraph data were merged by minute resulting in 1440 min per dog. The dogs’ 24-hr HRV (SDNN index) was calculated by PetPace. All dogs were in good health and none of the dogs were aggressive (i.e., all were approachable by Principal Investigator).

For proximity tagging, the Link monitor set as “receiver” records the relative quality of the Bluetooth signal from the Link device set as “beacon.” RSSI does not have an absolute value, and it does not correspond to a fixed spatial distance [21], although algorithms have be used to convert RSSI to distance within the first two meters [15]. In a validation study comparing location by wearable cameras and proximity tagging in office workers, the most accurate detection was when the participant was stationary [21]. It is important to note that ActiGraph recently (April 1, 2020) posted on their website that proximity data can be compromised when a single device is set to both receive and beacon simultaneously [22]. We have not used this option in the current study nor in our past study that utilized proximity tagging [15]. Moreover, our proximity data were verified to be accurate by a product support manager at ActiGraph.

Actigraph data from the monitors worn by both human and dog were downloaded using the Low-Frequency Extension [23] option and screened for wear-time using the graphing function in ActiLife. Cut points (axis 1 counts per minute, cpm) were set at sedentary (<100 cpm), light (100–1951 cpm), moderate (1952–5724 cpm), vigorous (5725–9498 cpm) and very vigorous (≥9499 cpm) for humans [24]. Cut-points (vector magnitude cpm) were set at sedentary (0–1351 cpm), light-moderate (1352–5695 cpm), and vigorous (>5696 cpm) for the dogs [25]. Participants’ sleep records were verified against sleep periods detected by ActiLife using the Cole-Kripke algorithm [26].

For the humans, position data from the ActiGraph thigh-worn monitor (lying/sitting vs. standing vs. stepping; 1-sec epoch) were combined with the position data from the chest-worn ECG monitor (lying vs. resting vs active; 1-sec epoch) to determine final position per second (Table 1). The camntech devices record movement as “active.”

For the dogs, position data collected via the PetPace Collar (sitting, standing, eating/drinking, lying on the back, lying sternally, lying right, lying left) were converted as follows. All lying variables were coded as “0”, sitting as “1”, and standing and eating/drinking as “2.” When more than one position was determined during a 60-s period, the position with the most seconds/minute was used.

Data from the ECG monitors were transferred to Actiwave Cardio Viewer and Actiheart software for initial analysis. Kubios HRV Premium v 3.3.1 software was used to further analyze 24-hr and minute-by-minute (1400 × 60-s epochs) ECG data [27]. The automatic artifact correction algorithm was applied to all data [28]. HRV data (60-s epochs) were merged with proximity, activity, and position data so that all data were lined up per minute throughout the 24-hr period, ensuring that HRV metrics were calculated from epochs of similar lengths [29]. The data were stratified by position (lying, sitting, standing), and then by proximity to the dog (RSSI absence or presence). The absence of an RSSI signal was identified as “absence of dog” and the presence of the RSSI signal was identified as “presence of dog”. How close the human and dog were to each other was not quantified; however, the presence of an RSSI signal would be indicative of the human (receiver) and dog (beacon) being in the same room (i.e., not separated by walls), whereas the lack of an RSSI signal would be indicative of the human and dog being in separate rooms (i.e., separated by wall or walls). The following time points were removed before final minute-by-minute data analysis: ≥ 2% artifacts, sleep, away from home, and interacting with another person (visitor) in the home. The different position/proximity combinations for the human–dog dyad are provided in Table 2.

The definitions for the following HRV time domain, frequency domain, and nonlinear parameters presented in the results section are provided in Table 3.

Analyses were performed using SigmaXL v8.1 and DataDesk v8.1 software. Heart rate, stress index, and all HRV parameters were tested for normality using the Anderson-Darling Normality test. Comparisons of human stress index, SDNN and rMSDD between RSSI absence and RSSI presence, and between positions, were tested using Wilcoxon signed rank test. All other comparisons (human variables: heart rate (HR), RR, very low frequency (VLF), low frequency (LF), high frequency (HF); dog variables: VVTI, pulse) were tested using paired *t*-test. Pearson correlations were used to test relationships between activity data generated from the ActiGraph monitor, ECG monitor, and PetPace Collar. All data are presented as mean ± SD, with statistical significance set at *p* ≤ 0.05, using 2-tailed probability.

## 3. Results

All participants completed the study. The human participants (3 men, 8 women; 2 African-American, 9 Caucasian) ranged in age from 56 to 83 years (Table 4) and BMI from 23–57 kg/m^2^. Average 24-hr SDNN index was 43 ± 18 ms, comparable to values reported from the Cardiovascular Health Study in older adults [8]. Nine of the eleven participants were current foster caregivers, and two of the participants had adopted their foster dogs. The participants spent between 54 and 94% time in sedentary behavior (Table 4). The relationship between percent time spent inactive (sum of lying, sitting, and standing) vs. percent time spent in sedentary behavior was significant (r = 0.90, *p* < 0.0005).

The dogs ranged in age from young to senior and spent between 77 and 93% time in sedentary behavior (Table 5). The dog breeds included American Eskimo (n = 8), Papillon (n = 1), Yorkshire Terrier (n = 1) and mix-breed Terrier (n = 1). None of the dogs in this study were included in the previous study on the effects of proximity between foster caregivers and their dogs on the dogs’ HRV [15]. There were no signs of adverse effects (e.g., allergic response, distress) of the collars on the dogs, and they all tolerated them without issue.

The relationship between percent time spent inactive (sum of lying, sitting, and standing) vs. percent time spent in sedentary behavior was significant (r = 0.94, *p* < 0.00005).

**Figure 1:**
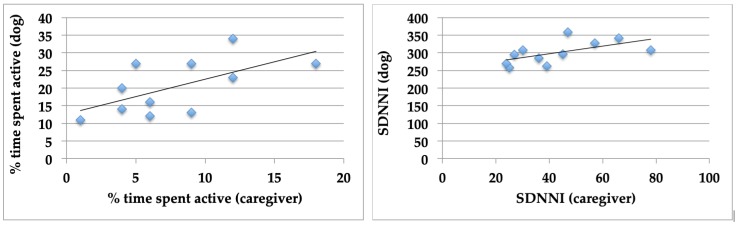
Relationship between activity (**left panel**) and HRV (SDNN index) (**right panel**) in the human–dog dyads over a 24-hr period.

The percent time spent active in the caregivers and dogs was related (r = 0.62, *p* < 0.05) (Figure 1, left panel), as was the SDNN index (r = 0.60, *p* = 0.05).

Human HR and HRV data by position and proximity (absence vs. presence of dog) are shown in Table 6. All data were normally distributed except for SDNN and rMSSD. Stress index was lower in the caregivers while (a) inactive (lying + sitting + standing combined) and (b) sitting in the presence vs. absence of their dogs. SDNN was higher in the caregivers while (a) inactive, (b) lying, (c) sitting, and (d) standing in the presence vs. absence of their dogs. rMSDD was higher in the caregivers while (a) inactive, (b) lying, and (c) sitting in the presence vs. absence of their dogs. VLF was higher in the caregivers while sitting, and HF was higher in caregivers while inactive in the presence vs. absence of their dogs.

Stress index, heart rate, and RR were higher in the caregivers while sitting and standing compared to lying. rMSSD and HF were lower in the caregivers while sitting and standing compared to lying.

The dogs’ pulse was significantly higher in the presence of their humans (absence vs. presence, 66 ± 8 vs. 70 ± 9 bpm, *p* < 0.005, n = 11). Heart rate variability (VVTI) was not significantly different when the dogs were in the presence of their humans (absence vs. presence, 11.41 ± 0.23 vs. 11.33 ± 0.20, *p* = 0.16, n = 11). There were not enough pulse and HRV data to calculate the differences in the dogs while in the absence and presence of their humans while sitting and standing.

Caregivers spent on average 50% of their waking time (while home) in the presence of their dog (absence vs. presence of RSSI, 469 ± 233 vs. 458 ± 193 min). Caregivers were almost twice as active when in the presence of their dog (absence vs. presence of RSSI, 6.8 ± 5 vs. 12.2 ± 6 steps/min, *p* = 0.02; 230 ± 205 vs. 372 ± 184 axis 1 cpm, *p* < 0.06) (sleep time and time away from home removed before analysis).

The positive and negative aspects of fostering a companion dog were captured with a modified DOSQOL questionnaire [19]. The scale is 1–7; 1 = strongly disagree and 7 = strongly agree. The results are shown in Table 7.

## 4. Discussion

A few of the many ways that the presence of a companion animal may impact emotional and physiological responses in humans include buffering stress response [31], lowering blood pressure [13], and improving autonomic function [32]. In the current pilot study, using novel proximity tagging technology, we show that stress index was lower, and HRV parameters (SDNN, rMSSD, HF) were higher when caregivers were in the presence of their dogs and inactive (lying, sitting, standing combined), as well as when in the presence of their dogs while sitting, vs. away from their dogs, over a 24-hr period. We also show that rMSDD and HF, two HRV parameters often reported in human-animal interaction research, were affected by body position, supporting the recommendation that posture, along with activity level, should be taken into consideration while measuring long-term HRV [33].

The presence of an unknown friendly dog has been shown to decrease LF/HF in dog owners [34] and to increase HF power in nonowners [9]. In the study by Kingwell et al. [34], 35 dog owners were seated for 10 min while near an unknown dog. The decrease in LF/HF was due primarily to a lower LF peak in the presence of the dog [34]. In the current study, the caregivers’ LF was not significantly affected while sitting in the presence of their dogs; however, HF power tended to increase and VLF significantly increased in the caregivers while sitting in the presence of their dogs. The HF band reflects parasympathetic activity; lower HF power is correlated with stress, panic, anxiety, and worry [29]. Normal VFL power indicates healthy function, and increases in resting VLF power may reflect sympathetic activity [35]. Low VLF power has been associated with inflammation, arrhythmic death, and PTSD (reviewed in [35]). In the study by Motooka et al. [9], four participants interacted with an unknown dog for two 30-min periods over a six-hour recording period in their home; neither activity level nor body position were documented. The HF power was significantly higher while the participants were near the dog vs. away from the dog [9]. Similarly, in the current study, HF power was significantly higher in the caregivers while inactive and in the presence vs. absence of their dogs.

Two studies have compared 24-hr HRV parameters between older pet owners and nonowners [11,12]. HRV recordings over 24-hr periods are considered the ”gold standard” for clinical HRV assessment; SDNN reflects the ebb and flow of all the factors that contribute to HRV and predicts morbidity and mortality, while the rMSSD estimates vagally-mediated changes reflected in HRV [29,35]. In post-myocardial infarction patients, 31 pet owners had higher 24-hr and daytime rMSSD compared to 71 nonowners [11]. In dog owners (n = 22) and nonowners (n = 80), 24-hr and daytime SDNN were also higher in the dog owners [11]. In individuals with one or more cardiac risk factors, 82 pet owners had higher 24-hr, day, and night rMSSD compared to 109 nonowners [12]. Although our experimental design was different from these two studies, we found that short-term SDNN and rMSSD were higher when the caregivers were in the presence vs. absence of their dogs while inactive (all positions combined), lying, and sitting; SDNN was also higher in the caregivers while standing in the presence of their dogs. The increase in short-term HRV while in the presence of a dog throughout the day likely contributes to the higher 24-hr HRV in pet owners reported in these two studies. Future research that combines 24-hr and minute-by-minute HRV before and after bringing a dog into the home (either short-term or long-term) could help to tease out the contributions of proximity, activity, and body position to overall autonomic function in both humans and dogs.

In the current study, the dog’s pulse was higher while in the lying position in the presence vs. absence of their caregiver. In our previous study on the effects of closeness and caregivers’ presence on dogs’ health outcomes using minute-by-minute proximity tagging, we found that the dog’s pulse was higher in the presence of their caregiver during sedentary bouts but not when in the lying position [15]. In the current study, the caregiver’s heart rate was not affected by the presence of the dog while the caregiver was lying, sitting, or standing. Similarly, there was no effect of the presence of an unknown dog in dog owners, while seated, on heart rate [34]. In the current study, the dog’s HRV, while lying, was not significantly affected by the absence or presence of the caregiver. In our previous study, we found that the dog’s HRV was lower in the presence of their caregiver during sedentary bouts but not when in the lying position [15]. The discrepancies between the pulse and HRV results in the current and previous study could be explained by (a) different set of dogs in each study (no dogs were included in both studies) and (b) the number of time points analyzed (data collected over one 24-hr period vs. 10–15 days). As each human–dog dyad is unique, it is not surprising that the dogs’ responses were not exactly replicated between studies.

Although there was a wide range in ages and activity levels in the caregivers and dogs, the percent time spent active over the 24-hr period was related in both members of the dyad. Similarly, SDNN index (24-hr measure) was related in the caregivers and their dogs. The SDNN index reflects combined sympathetic and vagal activity, and higher values are considered healthier [8]. Exercise training increased SDNN index in patients after myocardial infarction [36]. In a presented but unpublished study, we found that 24-hr SDNN index, rMSSD, SDNN, and HF were related to moderate physical activity in an older foster dog caregiver with neuromuscular disorder over a six-month period (r = 0.77, *p* < 0.05, n = eight 24-hr readings) [37]. The positive influence that dogs have on their owner’s physical activity is well documented (reviewed in [32]), and in patients with healed myocardial infarcts, 24-hr SDNN index is higher in dog owners than in nonowners [11]. A longitudinal One Health study design could help to determine whether these results reflect an interconnection between the caregiver and dog [17]. For example, if a sedentary person cares for an active dog, what happens to activity levels over time in each member of the dyad (controlling for HRV)? If a person with low SDNN index cares for a dog with high SDNN index, what happens to HRV over time in each member of the dyad (controlling for activity)?

The positive effects of being in the presence of a companion dog, i.e., decrease in stress index and increase in HRV parameters, were corroborated by the responses to the modified DOSQOL questionnaire. The potential positive aspects of dog ownership on quality of life, specifically provision of love, affection, and companionship, received high scores; the potential negative aspects, specifically stress induction, received low scores.

Limitations of the current pilot study include a small sample size and data collection during only one 24-hr period. Measuring 24-hr and minute-by-minute HRV parameters and physical activity in the human–dog dyad longitudinally and under different conditions (e.g., before vs. after adopting a dog, short-term vs. long term relationship between dyad) would provide more meaningful information on the effect of the human-animal interaction on these robust biomarkers, and help better understand the contributions of activity and proximity on 24-hr HRV metrics. A longer data collection time (more data points) would also allow for better estimations of the effects of proximity within 2 meters (closeness) on HRV in the dyad vs. only in the presence (RSSI signal) or absence (no RSSI signal) of a dog as in the current study. Another limitation is that the relationships between the dyads were not necessarily similar, other than the fact that all humans were either currently fostering a dog or had in the past, and that all dogs were either currently, or formerly, foster dogs. In addition, the quality of the relationship between the human and dog was not captured using an appropriate measure of the human-animal bond. These limitations can be properly addressed in a longitudinal study with a larger sample size and appropriate questionnaires to measure the bond.

## 5. Conclusions

The results of our pilot study demonstrate the usefulness of combining proximity tagging with ECG and activity monitoring to analyze the effects of human-animal interaction on minute-by-minute autonomic function during the daily lives of caregivers and their dogs. The findings also support the hypothesis that time spent in proximity to a companion dog throughout the day contributes to overall improvement in 24-hr autonomic function in foster dog caregivers. The potential for capturing the interconnection between the human–dog dyad, as shown by the relationship between the caregivers’ and dogs’ 24-hr HRV, should be addressed in future studies.

## Figures and Tables

**Table 1:** Determination of position using data from two accelerometers.

Final Position	Chest-Worn Accelerometer *	Thigh-Worn Accelerometer **
Lying	Lying	Sitting/Lying
Sitting	Resting	Sitting/Lying
Standing	Resting or Active	Standing
Active	Active	Stepping

* Cardio Viewer and ActiHeart 5 software (camntech); ** ActiLife software (ActiGraph).

**Table 2:** Position (of human participant) and proximity (absence or presence of dog) combinations for the human–dog dyad proximity measures.

Position	Proximity
Inactive *	absence of dog
Inactive	presence of dog
Lying	absence of dog
Lying	presence of dog
Sitting	absence of dog
Sitting	presence of dog
Standing	absence of dog
Standing	presence of dog

* Lying, sitting, and standing combined.

**Table 3:** Heart rate variability measures.

Time Domain:
Stress Index (SI): square root of Baevsky’s stress index [30] RR: time interval between successive ECG R-waves
SDNN: standard deviation of normal-to-normal RR intervals
SDNN index: mean of the standard deviation of RR intervals in 5-min segments over a 24-hr period
rMSSD: root mean square of successive RR interval differences
**Frequency Domain**:
VLF power: very low frequency (HRV frequency band set at 0–0.04 Hz) LF power: low frequency (HRV frequency band set at 0.04–0.15 Hz)
HF power: high frequency (HRV frequency band set at 0.15–0.4 Hz)

**Table 4:** Human caregivers: position, activity, and HRV over 24-hour period.

Dyad	Gender	Age (years)	ActiGraph + Camntech				ActiGraph				SDNN index (ms)
			% Lie	% Sit	% Stand	% Active	% Sed	% Light	% Mod	% Vig	
1	M	72	81	9	6	4	80	19	1	---	78
2	F	56	35	38	15	12	65	33	2	---	27
3	M	61	82	12	2	4	70	28	2	---	30
4	F	58	39	42	10	9	73	20	7	---	47
5	F	66	29	19	34	18	54	42	2	2	45
6	F	83	58	13	20	9	75	24	1	---	57
7	M	67	55	17	16	12	63	22	10	5	66
8	F	61	36	48	10	6	82	17	1	---	39
9	F	70	73	8	14	5	79	21	---	---	25
10	F	58	94	4	1	1	94	6	---	---	36
11	F	64	50	36	8	6	78	21	1	---	24

**Table 5:** Dogs: position, activity, and HRV over 24-hour period.

Dyad	Gender	Age * (years)	Time with Caregiver	PetPace Collar				ActiGraph		SDNN index (ms)
				% Lie	% Sit	% Stand	% Active	% Sed	% Light-Mod	
1	FS	12	2 weeks	80	3	3	14	91	9	308
2	FS	8	3 weeks	54	6	6	34	77	23	295
3	MN	1	2 weeks	76	3	1	20	87	13	308
4	MN	5	7 months	60	7	6	27	82	18	358
5	MN	12	10 weeks	71	1	1	27	83	17	296
6	MN	6	4 weeks	83	3	1	13	93	7	328
7	FS	12	11 months	66	4	7	23	89	11	341
8	MN	12	>1 year	78	2	4	16	88	12	262
9	MN	12	>1 year	59	8	6	27	82	18	258
10	FS	12	>1 year	86	1	2	11	91	9	285
11	MN	12	5 months	87	<1	<1	12	91	9	269

FS, female spayed; MN, male neutered; * approximate age.

**Table 6:** Minute-by minute human HR and HRV data (mean ± SD) by position and proximity.

Position	Lying + Sitting + Standing			Lying			Sitting			Standing		
Proximity signal	Absent	Present	*p*	Absent	Present	*p*	Absent	Present	*p*	Absent	Present	*p*
Dyad n	11			10			9			7		
Stress Index	23 ± 12	21 ± 11	0.04	21 ± 12	20 ± 12	0.06	25 ± 11 ^c^	23 ± 11	0.05	24 ± 12 ^b^	22 ± 11	
HR (bpm)	77 ± 16	76 ± 15		71 ± 13	70 ± 13		81 ± 18 ^b^	80 ± 17 ^c^		82 ± 19 ^d^	82 ± 16 ^e^	
RR (ms)	822 ± 181	826 ± 176		883 ± 169	886 ± 172		783 ± 197 ^b^	793 ± 197 ^d^		771 ± 197 ^e^	770 ± 176 ^e^	
SDNN (ms)	32 ± 43	34 ± 44	0.02	36 ± 46	38 ± 47	0.01	21 ± 22	23 ± 24	0.05	21 ± 15	22 ± 15	0.02
rMSSD (ms)	42 ± 71	44 ± 75	0.002	49 ± 75	51 ± 79	0.03	22 ± 31 ^b^	25 ± 33 ^b^	0.004	20 ± 17 ^b^	21 ± 18 ^a^	
VLF (ln)	3.7 ± 1	3.8 ± 1	0.10	3.8 ± 1	3.9 ± 1		3.6 ± 1	3.8 ± 1	0.04	3.8 ± 1	4.1 ± 1	
LF (ln)	5.2 ± 2	5.3 ± 1		5.2 ± 2	5.4 ± 2		5.1 ± 1	5.2 ± 1		5.2 ± 1	5.3 ± 1	0.08
HF (ln)	4.7 ± 2	4.9 ± 2	0.03	5.1 ± 2	5.1 ± 2		4.0 ± 2 ^c^	4.3 ± 2 ^b^	0.07	4.2 ±2 ^d^	4.3 ± 2 ^b^	

ln, natural logarithm transformed values of absolute powers of VLF, LF, and HF bands. a, *p* ≤ 0.10; b, *p* ≤ 0.05; c, *p* ≤ 0.01; d, *p* ≤ 0.005; e, *p* ≤ 0.001 vs. lying

**Table 7:** Modified Dog Owner Specific Quality of Life (DOSQOL) questionnaire responses (n = 11).

Fostering a Dog:	Mean ± SD
Provides me love and affection	6.9 ± 0.3
Provides me companionship when I want it	6.9 ± 0.3
Provides me emotional support	6.8 ± 0.4
Improves the amount of social activities I perform	6.2 ± 1.4
Improves my ability to do things for fun outside my home	5.8 ± 2.1
Improves my level of physical activity	6.5 ± 0.9
Interferes with my other household responsibilities	1.5 ± 1.2
Results in damage to my belongings or property	1.5 ± 1.2
Interferes with my ability to go on vacation or leave my house	2.2 ± 1.7
Increases my level of stress	1.3 ± 0.9
